# Social worlds of Appalachian women caregivers of older relatives living with dementia

**DOI:** 10.3389/fgwh.2024.1461626

**Published:** 2024-12-05

**Authors:** Brandy Renee McCann, Karen A. Roberto, J. Savla, Rosemary Blieszner

**Affiliations:** ^1^ Center for Gerontology, Virginia Tech, Blacksburg, VA, United States; ^2^ Center for Gerontology and Institute for Society, Culture and Environment, Virginia Tech, Blacksburg, VA, United States; ^3^ Center for Gerontology and Department of Human Development and Family Science, Virginia Tech, Blacksburg, VA, United States; ^4^ Human Development and Family Science, Virginia Tech, Blacksburg, VA, United States

**Keywords:** dementia, Appalachia, pandemic (COVID-19), activity restriction, respite

## Abstract

**Rationale:**

Over 11 million people in the United States provide care for an older family member with dementia, with this responsibility primarily falling on daughters and wives. In Appalachia, a mountainous region in the U.S characterized by close families, family members were crucial to ensuring that care needs were met for people living with dementia during the COVID-19 pandemic. However, we know little about the well-being of family caregivers during the public health crisis. Guided by a Limited Future Time Perspective postulate, which posits that as people age they begin to prioritize emotionally meaningful relationships over instrumental goals, we asked how dementia caregiving changes the social lives of family caregivers situated within kin networks; and how a public health crisis (i.e., COVID-19 pandemic) affects caregivers who are already at risk for social isolation and feelings of loneliness.

**Methods:**

Participants were recruited from a regional health care system and four Area Agencies on Aging. In our longitudinal study we invited family caregivers to be interviewed at multiple time points over a 4-year period. The sample for this study was women caregivers interviewed (*N* = 27; age range 32–81, *m* = 63). Interviewers followed a semi-structured protocol with questions designed to elicit descriptions about (a) changes in formal and informal support over time, (b) the person living with dementia's symptoms and disease progression, and (c) how the pandemic affected caregivers’ and persons living with dementia's social worlds.

**Findings:**

We found three types of caregivers: (1) caregivers who had social lives interdependent with their relative with dementia, (2) caregivers and persons living with dementia whose social lives were restricted due to dementia symptoms and caregiving demands, and (3) caregivers and their relative living with dementia who maintained separate social lives. Dementia symptoms more than social distancing measures contributed to caregivers’ shrinking social worlds particularly for those with interdependent social lives despite living amongst kin.

**Conclusions:**

This study is important in understanding how women in Appalachia fared during a pandemic in the context of dementia caregiving. This research supports the need for respite services and dementia care training for respite workers.

## Introduction

Approximately 11.5 million people in the United States care for a family member with dementia (1). In Appalachia, a mountainous region in the eastern United States, specific prevalence rates for dementia are unknown, but estimates for rural counties are higher than for urban counties and are expected to grow (2). Moreover, Appalachian people have disproportionately more risk factors associated with dementia (3), and like everywhere else, women are more likely to be engaged in dementia caregiving than men (4). Thus, becoming a caregiver for a relative living with dementia is a common life event for women in the Appalachian region of Virginia.

Family caregiving has many benefits and challenges. On the one hand, many family caregivers report positive aspects of their role including opportunities for increased closeness and sharing with their relative living with dementia (5). Conversely, long term caregiving can be challenging for family members who provide intense support, putting them at risk for psychological and physical health problems, including an increased risk of cognitive decline, heart disease, chronic loneliness and other conditions that disproportionately affect Appalachian people (6–8). Because rural, older Appalachian people typically take a wholistic view of health (9), social support and opportunities for social engagement may act as a buffer to the demands of caregiving and create avenues for resilience.

The purpose of this mixed methods study was to understand the social lives and feelings of loneliness among Appalachian family caregivers living in southwestern Virginia. In addition, we examined how an unusual external event, specifically the COVID-19 pandemic, impacted dementia caregivers' social worlds.

## Background

Family caregivers help their relatives with many instrumental activities of daily living (IADLs), including tasks like shopping and planning meals, communication with others (e.g., making calls), and transportation. Supporting these IADLs is important for people living with dementia because the cognitive skills needed to initiate and perform tasks such as using a phone and driving are critical to maintaining social connections, such as meeting a friend for lunch (10).

When a person living with dementia (PLwD) begins to need help with IADLs, a family member, typically a spouse or adult child, helps with or takes on these tasks (11). Family caregivers often recognize the importance of keeping their relatives living with dementia engaged in social activities and often put their own social lives on hold during their caregiving journey (12, 13). Depending on the cognitive symptoms of the PLwD (e.g., saying inappropriate things in public places) this change in social worlds may cause family caregivers to feel lonely if such changes isolate them from others (14).

Research on the social lives of family caregivers of people living with dementia typically focuses on network composition and, to a lesser extent, on changes in formal and informal care providers over time (15). Contrary to expectations, most caregivers and PLwD do not experience sudden social isolation but rather social interactions ebb and flow as symptoms change and helpers emerge and disappear. For example, Friedman et al. (16) study of a representative sample (*N* = 3,451) of family caregivers of PLwD found that caregivers experienced fluidity in their networks across time and especially during the COVID-19 pandemic. While most social networks remained stable throughout the pandemic with 61% of caregivers reporting “no network change”, 6% reporting “adapted” networks, 12% reporting “expanded networks”, and 21% of caregivers reported that their networks had “contracted”. Specific to dementia care support, caregivers also reported that their relative living with dementia moved to and from care facilities and caregivers were likely to use home and community-based services [HCBS] before the pandemic or begin using HCBS during the pandemic. This finding suggested that the social worlds of families affected by dementia may be increasingly fluid as care needs change over time. In a related paper from the same data set with a more focused subsample of caregivers who continuously provided care before and during the pandemic (*n* = 1,876), Kirkegaard et al. (17) reported that loneliness, among other caregiver well-being measures, was positively associated with increasing care provided over time. The authors suggested this relationship may be reflected in caregivers experiencing anticipatory grief as care recipients needed more care. Other researchers theorize that feelings of ambiguous loss (i.e., when a caregiver feels that their relative with dementia is psychologically absent despite their physical presence) characterize caregiving relationships due to dementia progression (18).

Restricted social worlds can have profound consequences for caregivers. A review of the literature of cardiovascular disease risks and caregiving suggested that having opportunities for leisure activities and social support were important protective factors (19). Strange et al. (20) found that spiritual engagement (e.g., going to church) strengthened kinship ties and intergenerational connectedness of middle-aged and older adults in Appalachia. They argued that the benefits of spiritual engagement staved off loneliness and enhanced people's sense of purpose. The risks of loneliness may be especially striking in the Appalachian region where Keefe and Curtain (21) point out that people rely on their kin networks and spiritual resources for both practical and emotional help. While health problems related to caregiving are prevalent in Appalachia in general (22), the region is also characterized by dense kin networks (23) that may buffer the negative effects of activity restriction associated with caregiving and provide social resources for dementia family caregivers.

In addition to informal support, HCBS use, particularly services that provide respite such as adult day care and personal care workers who also provide companion care in the home, are designed to give family caregivers a break from caregiving. How people use their respite time varies depending on how much respite they receive. Some caregivers use respite services primarily when they are traveling; others may use respite services so that they are able to maintain their employment. Caregivers also may use their respite time for running errands or attending regular social activities such as church or local recreational events. Despite the importance of respite services for caregiver well-being, HCBS use remains low in rural Appalachia due to an array of complex factors including a dearth of nearby services (24, 25), challenges in accessing services (26), and a shortage of affordable, high-quality respite workers (27).

Moreover, incongruence of care preferences between family carers and PLwD plays a role in caregivers' ability to take advantage of respite opportunities (28). In a study of care preference congruence across three domains—IADLs, personal ADLs, and socioemotional needs—Shelton et al. (29) found that particularly for socioemotional needs (i.e., companionship, activities, going out, emotional support), misalignment between caregiver and PLwD needs predicted poorer outcomes for caregivers. It is unclear whether being situated in the context of a rural area such as Appalachia where caregiving women often have multi-generational ties helps align care preferences of caregivers and their relatives with dementia.

## Theoretical lens

Socioemotional Selectivity Theory posits that as people get older, they take a limited time perspective (i.e., an awareness of the nearness of the end of the life span) and become more focused on emotionally rich goals (30). In response to the COVID-19 pandemic, we wondered how caregivers' social goals might change because of social distancing restrictions. Additionally, given that dementia is typically a long, degenerative condition, we recognized that caregivers and PLwD might prioritize social relationships over safety goals. However, family caregivers and PLwD may have conflicting wishes regarding their social lives. For example, during the pandemic, if a PLwD experienced confusion in public places or feared for their safety, rather than going out to socialize they may have wished to have visitors at home only or have no visitors at all. In contrast, their caregiver may have wanted to continue to find ways to attend public gatherings while social distancing.

Guided by the research on a Socioemotional Selectivity Theory, in particular the limited time perspective, we sensitized our analysis in three areas: (1) for both dementia progression and the time of the pandemic there is a heightened possibility of death, both of self and close others; (2) the future is unclear and there is uncertainty about how long a caregiving situation will last and how the pandemic will unfold; and (3) goals and motivations are likely to be present-focused on meaningful relationships.

## Methods

**Figure 1:**
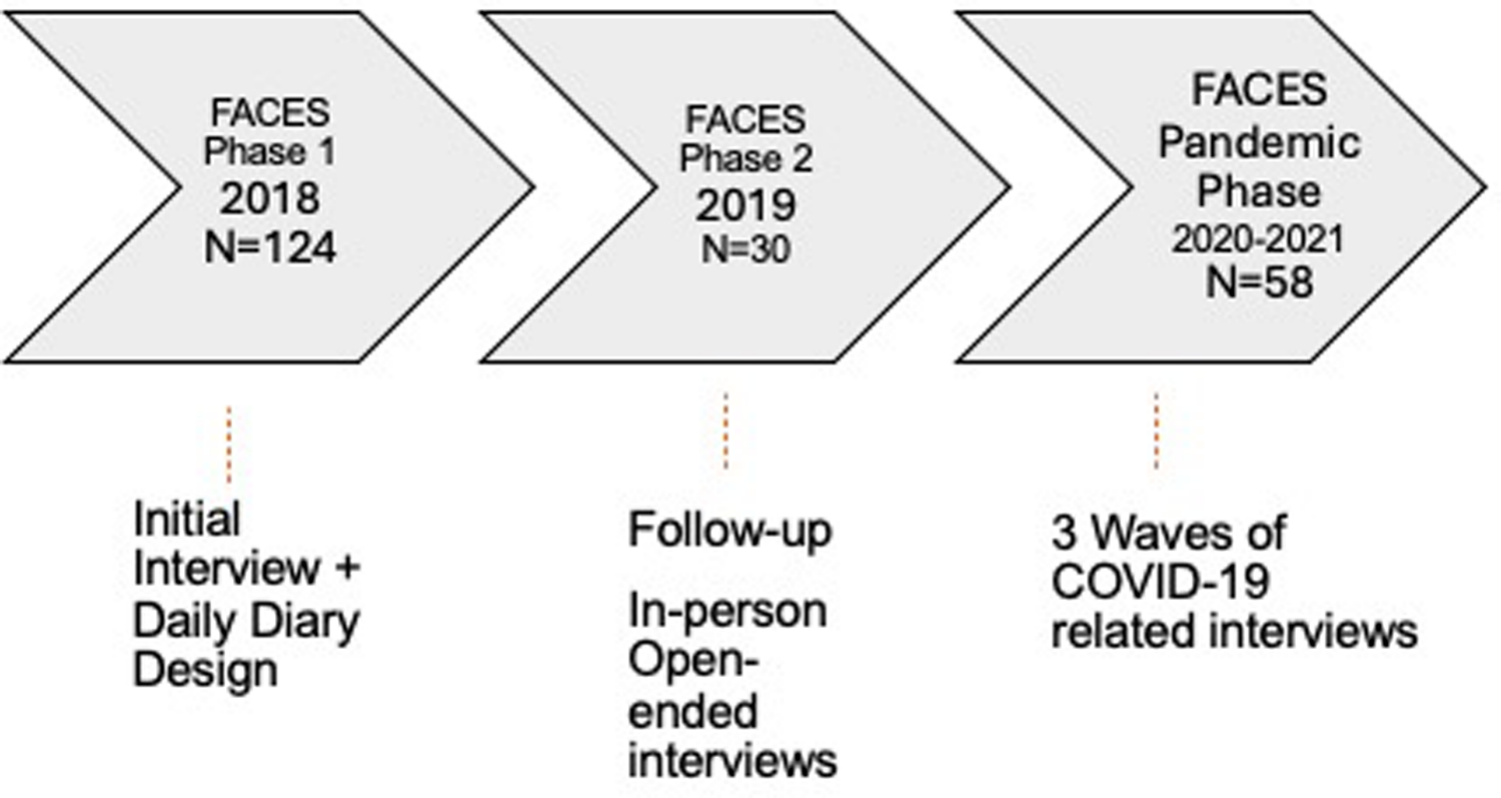
Participant interview timeline.

Data for this paper came from a longitudinal, mixed-methods study of Appalachia-residing family caregivers of persons living with dementia, called FACES-AD (Families in Appalachia Caring for Elders with Alzheimer's Disease; see Figure 1). Counties included in the study footprint were a mix of distressed, at-risk, or transitional economies as defined by the Appalachian Regional Commission (31) and were thus reflective of southern and central Appalachia in general. Initially, dementia family caregivers living in Appalachian Virginia were invited to participate in a telephone survey, followed by a 7-day diary focused on their caregiving experience: 124 participants took part in Phase 1 [see (32) for design details]. Approximately 2 years later we invited participants who were long-time Appalachian residents (+20 years) to participate in in-depth, in-person interviews [see (28) for sampling details]; 30 caregivers from Phase 1 took part in Phase 2.

Additional data collection took place during the COVID-19 pandemic. We invited all caregivers from Phase 1 to participate in three telephone interviews to share their pandemic experiences. Of the 117 caregivers in Phase 1 who agreed to be contacted for follow-up interviews, 58 agreed to take part in the first pandemic interview (of those who did not participate 30 PLwD had died, 20 caregivers were unreachable, and 9 caregivers declined). Twenty-two caregivers were spouses, 26 were adult children, 2 were sisters, and 3 were nieces. The first pandemic interviews took place in April 2020; the second pandemic interviews took place in June 2020; and the third pandemic interviews were conducted in January 2021.

The inclusion criteria for the current analysis were that caregivers had to be women and had to have participated in at least 3 of the 5 interviews (to be clear, all participants were interviewed in Phase 1 and the first pandemic interview + at least one additional interview). Most of the women caregivers lived in the same western Virginia town or county in which they were born; three of the women were inter-regional migrants who had moved from other places within Appalachia, most typically from the coal fields of West Virginia. To assess participants’ saturation in place and the likelihood that they would have extensive social networks, we asked how long they had resided at their current zip code and in what county and state they were born. We excluded anyone from analysis who had lived in their zip code for fewer than 20 years or who was not born in the greater Appalachian region as defined by the Appalachian Regional Commission. Thus, for this analysis, we restricted inclusion to caregivers who had been interviewed at least three times over the course of the project and who had been born and were currently residing in the Appalachian region (*N* = 27). Three caregivers, all of whom were daughters, were African American; the rest were white. The caregivers ranged in age from 32 to 81 (*m* = 63 years old). Fifteen women lived with their relative who had dementia; 12 did not co-reside.

This project was approved by the Institutional Review Board at Virginia Tech (#2284) and researchers obtained consent at each wave of the study. Pseudonyms are used and identifying details are changed.

## Measures

### Loneliness

To create a baseline measure of loneliness, we used the UCLA Loneliness Scale administered during Phase 1 of the study. Participants rated 3 items measuring subjective feelings of loneliness on a scale of 0–3 (i.e., how often do you feel lonely; how often do you feel left out; how often do you feel isolated from others). We summed scores for each participant with “0” meaning that participants “never” felt lonely and “9” meaning that participants “always” felt lonely.

During the three COVID-19 interviews, we asked caregivers several open-ended questions related to how closely they followed social distancing measures, how they were taking time for themselves, and whether they felt that the pandemic had affected their relationships with friends and family. Probes included following up to determine if reported changes in their social worlds were the result of the pandemic, the result of their caregiving role, or some combination.

### Social worlds

We used Interpretative Phenomenological Analysis [IPA; (33)] to investigate the interviews of the 27 family caregivers. IPA is a branch of phenomenological research and is suitable for questions that focus on meaning-making among participants who have a common experience, such as being a caregiver for a PLwD. Consistent with IPA, we began by open coding the data, then looked for common patterns or themes. Next, we began to cluster participants based on their experience. The first author read the caregivers' interviews, noting important aspects of their relationships with the PLwD, including shared activities as well as caregivers' social worlds more broadly. We used an Excel spreadsheet to keep track of changes over time, quotes, and notes in each phase of the study for each of the 27 families. The second author verified coding for each family. If the second author disagreed with the coding of the first author, we discussed the case until agreement was reached on social world designation.

## Findings

Caregivers and their relatives living with dementia tended to have stable social worlds over the course of the study with shifts focused on PLwD changes in cognitive or physical abilities. We found three types of caregiver social worlds: (1) caregivers who had a *separate* social world from PLwD in which caregivers maintained social lives independent of their caregiving responsibilities and the relative living with dementia had social outlets that did not rely exclusively on the primary caregiver; (2) caregiver social worlds that were *restricted*, in which family caregivers were the PLwD's primary or only social outlet and caregivers had little time for other kinds of social interactions; and (3) i*nterdependent* social worlds with PLwD, in which caregivers and PLwD regularly shared activities and company in ways that were mutually satisfying. Generally, the social lives of family caregivers of people living with dementia remained stable; when they changed it was a result of the progression of their relative's dementia rather than long-term social disruptions caused by the COVID-19 pandemic.

Below we present examples for each type of caregiver using details from the caregivers' interviews.

### Separate social worlds (*n* = 12)

Twelve family caregivers had separate social lives from the PLwD for the duration of the study. Caregivers in this group comprised 8 daughters, 1 niece, 1 granddaughter, and 1 wife. The caregiver and PLwD were an important part of each other's lives, but they each had other social outlets and important relationships. Not surprising, this type of dyad was characterized by lower levels of caregiver loneliness (sum scores ranged from 0 to 6) and the PLwD had mostly moderate symptoms and needed less intense care at the beginning of the study. These relationships were characterized by emotional closeness and concern on the part of the caregiver, with an added dynamic of help with IADLs (e.g., help with transportation or making plans).

For example, one daughter caregiver and her mother with dementia attended church together on Sundays and remained active in their congregation by attending church outdoors throughout COVID-19 shutdowns until the last pandemic interview when the mother with dementia could no longer focus on the service and refused to mask for the entire time. Even with this change, personal care workers and other family provided support and socialized with the mother during the study. Particularly, the personal care worker who was helping the PLwD only two days per week during Phase 1 had increased the time she spent with the mother to 40 h per week by the final interview. The daughter caregiver described the personal care worker as a “perfect companion” for her mother. Thus, even though the caregiving daughter said that she felt a lack of social opportunities during the pandemic, she also said she had “lots of time” to herself to socialize at church, go fishing with her husband, or to engage in solitary hobbies she enjoyed such as reading or taking walks.

Another daughter caregiver and her brother shared responsibility for their mother, with whom the brother lived. She saw her mother on most days and assumed the role of personal care worker and companion for her mother. She scheduled her time so that she could be available to help her mother with bathing twice a week and took her out of the house almost daily. Their favorite activity was eating out. During the pandemic interviews, the daughter said she had time to do the things she wanted to do such as spending quality time with her husband and taking a vacation; however, she felt guilty when she needed to be away, and have other family members cover for her. Explaining how they coped with not eating out during the pandemic, the daughter said: “[Before the pandemic, we] would go to eat out. […] At first [my mom would say], “Why don't we go here to eat”, [but] I think she's kind of gotten used to the fact that we can't do that […]. [On Sunday,] we went to Sonics and, of course, ate in the car, and then we took a ride. She's okay if you do that”.

A different daughter caregiver who also shared care responsibilities with a brother explained during Phase 2 interviews that caregiving was stressful because her mother, who lived alone in her own home, called her and her brother frequently. The mother called because she experienced confusion and occasional hallucinations and had a need for help to stay grounded in reality. The caregiver said, “I have a life, and it gets kind of stressful, trying to do what I want to do and take care of her at the same time. I don't want to be here just “on call” all the time”. The caregiver was in a new romantic relationship after years of widowhood and ensured that her brother was available for calls and check-ins during evenings when she had plans. This pattern continued throughout the pandemic until the mother passed away after a fall near the end of the study period.

The wife with a separate social life from her husband, notably a late life marriage, said: “[Dementia] makes it difficult to carry on a conversation. Sometimes it gets kind of frustrating because I feel like I don't have anybody to talk to. Because when I'm talking to him, he's just not paying attention”. Throughout the study the PLwD enjoyed socializing with his friends at an adult day services group. Even during the pandemic, during the period when they could not meet in person, the leaders of the group created a Facebook page where they could interact and have regular video chats. The caregiver enjoyed visiting her family on her own in another town when she had time to do so. Because their marriage occurred later in life, and the husband's dementia symptoms emerged early in the marriage, it is not clear to what extent this couple may have had interdependent social worlds prior to their caregiving journey.

### Restricted (*n* = 6)

Distinct from the women who had separate social worlds, family caregivers who were categorized as having a restricted social life described limited engagement in other relationships and activities because of their caregiving situations. They reported feeling moderate to high levels of loneliness. Four dyads were mother/daughter and two were husband/wife. One wife caregiver spent most of her time with her otherwise healthy and strong husband who had to be constantly monitored due to pronounced confusion. He was prone to wandering; he moved furniture around the house; and he needed help remembering to eat and drink. Their daughter helped when she could but was employed and had young children of her own to attend. Even though the wife caregiver spent a lot of time with her husband, she reported high levels of loneliness (sum score 8) and said of her husband, “[He's] just kind of in his own little world now”. This comment was typical of caregivers in the restricted category where the PLwD was in the latter stages of dementia progression and experienced confusion and pronounced memory loss.

A daughter in the restricted group who had respite workers twice per week said she used that time doing necessary chores and errands rather than socializing. She reported the lowest levels of loneliness of the restricted group (sum score 4) and said that her brother visited them almost daily. The brother did not stay alone with his mother to provide respite for his sister because the mother refused to allow men to help her with personal care. The caregiver received respite from two care workers whom she hired through word of mouth. She explained that her mother demanded her full attention and said, “I have to go out to maintain my sanity… On Mondays, I have somebody that stays five hours for that. That is for anything that I have to go out and get. And whether it be groceries or any kind of errands I have to run, that's what I do on Monday. Then on Fridays, with the other lady, I have to do all my yard work”. When asked if she was ever able to get time for herself during the pandemic, this daughter said, “No, my time out of the house […] I do what I've got to do and hurry up about it”.

Typical of families in the restricted category, family situations either remained static during the pandemic, or they lost in-home paid help furthering their restricted situation (32). A daughter caregiver, whose social world was restricted to her friendships with the aides who came to help with her mother's care, said that when social distancing restrictions began to lift, she faced more frustrations. She said, “People are scared to come into my house right now. I do [my mom's] nails, I do her toenails, and I am cutting her hair, which is not fun let me tell you. But, if I could get her the vaccine, then things would loosen up a bit. […] The girl that does her hair is a sweetheart, she's a good friend of mine, but she is terrified of coming in and killing my mother”. In this situation, the mother living with dementia was bedbound and at the time there was no plan in place in Virginia to vaccinate homebound people.

### Interdependent (*n* = 9)

Of the nine caregivers who described interdependent social worlds at the beginning of the study, five participants described social lives that were interdependent with their relative living with dementia for the study duration. Conversely, four participants had interdependent social lives at Phase 1, but changed over the course of study, with two describing more restrictive social worlds over time, and the other two caregivers discussing how they began living separate social lives. In general, caregivers with interdependent social worlds reported more varied levels of loneliness at Phase 1 (from 9 to 0) and were the only group with participants that moved to another social world category. Like caregivers in the restricted category, caregivers who had an interdependent social world had a challenging time meeting their own needs for social engagement if their relative with dementia became house bound.

Of the 5 caregivers and PLwD who had interdependent social worlds throughout the study, 3 were mother/daughters, 1 was husband/wife, and 1 dyad was sisters. The dyads tended to go to church together, and the caregivers consciously included the PLwD in the flow of the day including visiting with other family and running errands. One caregiver whose sister did not like to leave the house, felt that it was part of her responsibility to keep her sister socially engaged by encouraging others to visit even though the PLwD no longer recognized some family members. During the pandemic, this caregiver took her sister for car rides simply to get her out of the house. Because the caregiver had support of other family members, she was able to go to church on her own even if her sister refused to go, which was a major social outlet for the caregiver.

A daughter caregiver took her mom almost everywhere she went. At Phase 2 she reported looking for a part-time flexible work arrangement, such as data entry from her home, so that she could be with her mother while working. Although the caregiver wished for more alone time with her husband, she reported never feeling lonely (0 sum score). She and her mother spent their days going to church-related activities, from Sunday services to senior luncheons to volunteer opportunities. During the pandemic, they visited other family members in person but practiced social distancing. The caregiver felt that other than restricting their holiday plans during the winter of 2020, the pandemic had “no impact” on their lives. This highly interdependent dyad raises the question of what might happen to the caregiver's social world if her mother became resistant to their frequent outings.

Another daughter and mother started out in the study with an interdependent social life, but as time went on moved into the restricted group. They had lived together for decades beginning when the caregiver got divorced as a young woman and moved back in with her parents. The daughter gradually slid into a caregiver role once her father passed away. During the study, the daughter reported that they lived in a run-down apartment complex that the caregiver hoped to move out of. Neither woman drove. At the beginning of the study the caregiver's nephew lived with them and they relied on either him, friends, or a shuttle run by the local area agency on aging for transportation for shopping and other small errands, church attendance, or doctor appointments, respectively. By Phase 2, the nephew had moved out and the daughter reported feeling trapped in their small apartment with her mother who followed her from room to room throughout the day. Her only respite was when friends picked her up for church on Sunday mornings while other friends or her sister visited with her mother. This situation became more pronounced during the social distancing phase of the pandemic when churches no longer met in person and doctor's appointments were cancelled.

In contrast, another daughter-mother dyad, who started out as interdependent, gradually moved into the separate category during the study. Even though the PLwD did not often leave her home, this caregiver felt emotionally close to her mother, had a strong desire to help her mother, and visited almost daily. Importantly, this daughter shared care with her siblings, who all took turns staying with the mother in addition to having personal care workers. By the pandemic waves of the study the mother was receiving hospice and the caregiver said she and her siblings were spending less time visiting with her mom in part because of the pandemic (caregiver had a public-facing job) and partly because they had hired 24/7 care workers. She said, “[My mother is] really close to two of the women that stay with her”. Because she had trusted help whom her mother enjoyed, the caregiver could spend time gardening at her own home or enjoying visits with her own daughter and grandchildren.

Likewise, a wife caregiver said that she and her husband shared a social life at the first interview; the husband was still able to go out and do limited errands on his own. By the time of the COVID interviews, the caregiver was having physical health problems, and the husband's physical and cognitive problems had progressed such that he had moved into a skilled nursing facility. This married couple spoke on the phone daily and the caregiver visited him when possible. However, because the caregiver had a chronic autoimmune condition, she limited how much time she spent with others in person, including her husband. She attended an online church service and talked frequently with her daughter from a previous marriage. Of the caregivers who had separate social lives (either for the duration or as a change), this caregiver reported the highest level of loneliness (sum score 7) at Phase 1. Also worth noting, she was one of the most cautious caregivers we spoke with during the pandemic interviews, yet she was one of the few caregivers who contracted COVID-19 during the study.

## Discussion

The social worlds of Appalachian women caregivers of older relatives with dementia were complex. Through our interviews with older women caregivers prior to and during the COVID-19 pandemic we came to understand how caregivers of relatives living with dementia experienced shutdowns in the Appalachian region, potential disruptions to care, and social distancing. Generally, our research is congruent with other findings that show that the social worlds of older adults typically remained stable during the pandemic [e.g., (34)].

Additionally, our analysis supported a Limited Time Perspective, a central proposition of Socioemotional Selectivity Theory, with some caveats. First, analysis revealed three types of social worlds that caregivers and PLwD inhabited. Some had separate social lives and were able to maintain them throughout the duration of the study; others had restricted social lives in which family caregivers felt they not only had few opportunities for socializing outside the caregiving dyad, but that their relative had lost their capacity to maintain emotional reciprocity within their relationship due to their dementia symptoms. A third type of social world, interdependent, referred to dyads in which both the PLwD and their family caregiver enjoyed spending time together and enjoyed socializing. Notably, it was in this category that we saw change over time due to the progression of dementia symptoms and caregivers' prioritization of PLwD social desires.

A Limited Time Perspective typically focuses on an individual's social goals; our research suggests that being in a caregiver role, with the responsibility of helping a relative with memory problems maintain their social worlds, can create a situation where the caregiver experiences conflicting social goals. Importantly, the salience of this conflict in goals seemed to be independent of the study context, Appalachian Virginia (14). That is, as PLwD needed more assistance, caregivers either spent more time developing their own social world outside the dyad (interdependent to separate) because they had help, or their social world shrank along with that of their relative (interdependent to restricted).

Although all the women caregivers interviewed were embedded within regional kin networks, their experience of dementia caregiving and their feelings of loneliness tended to vary according to how interdependent, separate, or restricted their social worlds were. Typically, these caregiving family members entered their dementia journeys with interdependent or separate social worlds. As the PLwD symptoms progressed, family caregivers began to have an awareness of limited future time. They reorganized their social worlds to cope by either spending more time with their relative through joint social activities, hiring paid caregivers, enlisting other family members to help with caregiving tasks or provide respite, or by restricting their own social lives to focus on the needs of their relative.

Our findings align with recent research on loneliness and caregiving (14, 35) in that caregivers in our study with more restricted, isolated social worlds tend to report greater feelings of loneliness, particularly when there was a difference between what caregivers wanted to be doing vs. their reality. Future research is needed to identify the most effective strategies for maintaining well-being and coping with loneliness when caregivers face conflicting social goals (i.e., wanting to spend time with PLwD vs. wanting time to engage in other meaningful activities). This is especially important for those with restricted social lives who may choose these restrictions due to the belief that their time with their relative with dementia is limited.

Even with the conditions of a global pandemic, which imposed additional restrictions on caregivers and their relatives living with dementia, caregivers typically discussed dementia symptoms rather than the pandemic as detrimental to their social lives. Future research is needed to understand which particular dementia symptoms trigger a change in social worlds. Anecdotally, some caregivers mentioned that the development of apathy and withdrawal, inappropriate social behaviors such as saying rude things to others, or difficulties with managing incontinence led to changes in their social worlds. Targeting interventions to address specific dementia symptoms may improve social outcomes for primary caregivers, PLwD, and their families.

High quality respite care for caregivers and/or companion care for PLwD were important sources of support, particularly during the pandemic. Our findings support Hash et al. (6) call for providing more training and educational programs on aging and dementia care for direct care workers in Appalachia. Personal care and respite workers who provided companionship to PLwD were important for families' social-emotional well-being; this finding may be true for women and men living in other disadvantaged or geographically remote areas where opportunities for social engagement outside the extended family are few. Opportunities for maintaining relationships with people outside the caregiving dyad is an important aspect of caregiver resilience (12, 36). Moreover, although PLwD sometimes prefer the company of a primary caregiver as their memory declines and they recognize fewer people, we found evidence that PLwD formed close relationships with respite workers who regularly visit. This was especially important during the pandemic if the families lost other types of help [e.g., (32)].

Although our study was longitudinal, it is important to note that it did not cover the entire dementia caregiving journey from the beginning to end. Instead, we focused on a 3- to 4-year period of care, with caregivers typically entering our study when they were providing moderate levels of support. For women caregivers whose social worlds changed during the study, we found evidence in retrospective accounts of their dementia journeys that change in caregiver and PLwD's social worlds are likely to occur over time for most families. Thinking beyond the Appalachian region, future research should capture the evolving nature of the social worlds of caregivers PLwD and feelings of loneliness to better understand how changes impact their care decisions and well-being.

## Data Availability

The study data are not available because the primary investigators have not completed their original work with the data set. The study was not preregistered.
